# Pain-Related Factors and Their Impact on Quality of Life in Chinese Patients With Amyotrophic Lateral Sclerosis

**DOI:** 10.3389/fnins.2022.897598

**Published:** 2022-07-13

**Authors:** Ran An, Yuan Wu, Yi Li, Xin Li, Shaolong Ai, Yanming Xu, Chengqi He

**Affiliations:** ^1^Key Laboratory of Rehabilitation Medicine in Sichuan Province, Department of Rehabilitation Medicine, West China Hospital, Sichuan University, Chengdu, China; ^2^Department of Neurology, West China Hospital, Sichuan University, Chengdu, China

**Keywords:** pain, amyotrophic lateral sclerosis, ALS, QoL, quality of life

## Abstract

**Objectives:**

Pain is considered a common symptom in amyotrophic lateral sclerosis (ALS). However, the results of studies on pain in ALS are limited and inconsistent. The aim of our study was to comprehensively evaluate the potential factors of pain and effects on quality of life (QoL) in patients with ALS from China.

**Participants and Methods:**

Patients were eligible if they fulfilled the criteria of probable and definitive ALS according to the revised El Escorial criteria. Pain was assessed by the Brief Pain Inventory (BPI). Disease severity, sleep quality, fatigue, anxiety, depression, and quality of life (QoL) were evaluated in ALS patients by the ALS Functional Rating Scale-revised (ALSFRS-R) and ALS severity scale (ALSSS), Pittsburgh Sleep Quality Index (PSQI), Fatigue Severity Scale (FSS), Hamilton Anxiety Rating Scale (HARS), Hamilton Depression Rating Scale (HDRS) and McGill Quality of Life Questionnaire (MQOL). Then, the clinical characteristics of ALS patients with pain were compared with those without pain. Last, associated factors of pain, as well as impact on QoL in Chinese ALS patients, were assessed.

**Results:**

A total of 86 ALS patients were included. ALS patients with pain tended to have higher FSS scores and poorer QoL. The FSS score and ALSSS [lower extremity (LE) + upper extremity (UE)] were associated with pain in ALS patients. The ALS Functional Rating Scale-revised (ALSFRS-R), Pain Severity Index (PSI), HARS and HDRS scores were significantly associated with both the physical and psychological domains of QoL.

**Conclusion:**

Our study was the first to comprehensively evaluate factors associated with pain in Chinese ALS patients, finding that fatigue can be a risk factor for pain and ALSSS (LE + UE) score was related with pain intensity. Additionally, we identified the adverse effects of ALSSS (LE + UE), HARS and HDRS scores on QoL in Chinese ALS patients.

## Introduction

Amyotrophic lateral sclerosis (ALS) is a neurodegenerative disorder of unknown etiology characterized by the progressive loss of upper and lower motor neurons, causing weakness and atrophy of upper and lower limbs, dysphagia and dysarthria, which eventually result in death due to respiratory failure typically within 2–4 years of onset (Chiò et al., 2017). No effective treatment is available for ALS patients, so the predominant clinical management is symptom relief to improve quality of life (QoL) (van Groenestijn et al., 2016). Quality of life for ALS patients is affected not only by the physical aspects of the disease but also by the psychosocial effects of living with this terrible disease (Goldstein et al., 2002).

Pain is considered a common symptom in ALS, with frequencies varying from less than 15% to 85% (Newrick and Langton-Hewer, 1985; Chiò et al., 2012, 2017; Pagnini et al., 2012; Pizzimenti et al., 2013; Rivera et al., 2013; Wallace et al., 2014; Hanisch et al., 2015; Stephens et al., 2015; Moisset et al., 2016; Ishida et al., 2018; Lopes et al., 2018; Delpont et al., 2019; Gicalone et al., 2019; Taga et al., 2019; Åkerblom et al., 2020; Edge et al., 2020; Kong et al., 2021; Verschueren et al., 2021). Guidelines for ALS treatment reported that pain may be present in ALS patients and should be treated (Miller et al., 2009). Our previous study also identified that pain in ALS patients was common and insufficiently treated compared with healthy control subjects (An et al., 2021).

Disease duration, disease severity and depression were the common variables included to investigate pain and the influences on quality of life in patients with ALS (Lou et al., 2003, 2010; Chiò et al., 2004; Pagnini et al., 2012; Pizzimenti et al., 2013; Lopes et al., 2018; Edge et al., 2020). Studies about pain in ALS can be significantly meaningful given that pain might be related to distress, depression, and reduced mobility, which may contribute to a poorer QoL (Pizzimenti et al., 2013). However, studies on the impact of pain on QoL in ALS patients are limited, with conflicting results (Pagnini et al., 2012; Pizzimenti et al., 2013; Lopes et al., 2018; Edge et al., 2020). Some studies suggested that pain was associated with deterioration of QoL in ALS patients (Pagnini et al., 2012; Edge et al., 2020), while others indicated that pain intensity was not related to QoL (Pizzimenti et al., 2013; Lopes et al., 2018). Although the purpose of a previous study from a northern city of China aimed to evaluate the effect of pain on quality of life in ALS patients (Kong et al., 2021), however, there are obvious errors in their methodology section. In their study, pain-related interference with daily activities was conducted between ALS patients with pain and ALS patients without pain by the BPI questionnaire. Brief Pain Inventory (BPI), a multidimensional scale that provides basic information of pain, also includes the influence of pain on some domains of daily activities. Obviously, it seemed unsuitable to use BPI questionnaire to evaluate the impact of pain on daily functions in ALS patients without pain at all. Moreover, the lack of standard specialized questionnaires for measurements of other QoL-related confounding factors and QoL, such as HARS and HDRS scale for anxiety, depression and MQOL scale for QoL, may be another shortcoming (Kong et al., 2021). Therefore, further studies are urgently needed to verify the contradictory or inaccurate results at present.

Considering the potential impact of pain on QoL in ALS patients and their caregivers, the identification and evaluation of factors associated with pain and its impact on quality of life in ALS patients are of great importance. Therefore, we speculated that some motor or nonmotor symptoms of ALS patients may be associated with pain and that ALS patients with pain may have a much poorer QoL.

Accordingly, the objective of our study was to comprehensively evaluate the potential factors associated with pain and the impact of pain on QoL in ALS patients from China.

## Participants and Methods

### Participants

The study was conducted in West China Hospital of Sichuan University in Southwest China. All patients with ALS were recruited from November 2020 to August 2021. Patients were eligible and consecutively recruited if they fulfilled the criteria of probable and definitive ALS according to the revised El Escorial criteria (Brooks et al., 2000). We used the Mini-Mental State Examination (MMSE) to assess cognition in all patients. Patients were excluded if they had cognitive impairment or significant concomitant diseases preventing them from being able to select responses on self-report questionnaires.

### Methods

#### Pain Assessment

Pain was evaluated using the Chinese version of the Brief Pain Inventory (BPI), a multidimensional scale (Wang et al., 1996). Brief Pain Inventory is a structured self-administered qualitative and quantitative questionnaire that provides basic information of pain in the last week, indicating the worst, least, average pain intensity as well as the pain perceived at the time of the interview (scale from 0, “no pain,” to 10, “pain as bad that you can imagine”). A Pain Severity Index (PSI) was derived by averaging the following pain severity items: worst and average pain and pain perceived at the time of the interview (Hoffman et al., 2010; Chiò et al., 2012). The degree of pain was defined as no pain (PSI = 0), mild pain (1 ≤ PSI ≤ 3), moderate pain (4 ≤ PSI ≤ 6), and severe pain (7 ≤ PSI ≤ 10) (Chiò et al., 2012; Hanisch et al., 2015; An et al., 2021).

#### Physical Functional Status and Disease Severity Assessments

The physical functional status of ALS patients was evaluated with the ALS Functional Rating Scale-revised (ALSFRS-R), a 12-item scale. Each item is rated from 0 (worse) to 4 (best), corresponding to a total score ranging from 0 to 48, with higher scores indicating greater physical status and function (Cedarbaum et al., 1999).

The amyotrophic lateral sclerosis severity scale (ALSSS) has been developed to provide a means of rapid functional assessment for patients with ALS. The scale included four categories of speech (SP), swallowing (SW), lower extremity (LE), and upper extremity abilities (UE). Each section has ten possible rating scores (1–10) based on the progressive decline of function. The bulbar function [ALSSS (SP + SW)] can be described by adding the scores for SP and SW. Similarly, spinal function [ALSSS (LE + UE)] can be described by adding the scores for LE and UE. A total score can be assigned by adding the four subscores (SP + SW + LE + UE) (Hillel et al., 1989). Other clinical data, such as sex, age, age at onset, site of onset, and disease duration, were collected from all the ALS patients included.

#### Sleep Quality and Fatigue Assessments

Sleep quality was assessed using the Pittsburgh Sleep Quality Index (PSQI). A PSQI score > 5 indicates poor sleep (Buysse et al., 1989). Fatigue was evaluated by the Fatigue Severity Scale (FSS), a self-report questionnaire with nine items that measures subjective fatigue in the past 2 weeks. Subjects were asked to rate the degree of fatigue on a scale of 1 (strongly disagree) to 7 (strongly agree). The average score of the nine items was recorded as the FSS score, with a higher FSS score suggesting a higher degree of fatigue intensity. Fatigue was defined as a FSS score ≥ 4 (Wang et al., 2016).

#### Depression and Anxiety Assessments

Depression and anxiety were assessed using the 24-item Hamilton Depression Rating Scale (HDRS) and 14-item Hamilton Anxiety Rating Scale (HARS), respectively. The HDRS consists of 24 items; the severity of each item is rated from 0 (not present) to 4 (very severe) or 2 (present), and the scores range from 0 to 76 (Moberg et al., 2001). The HARS included 14 items, the symptom severity of each item was rated from 0 (not present) to 4 (very severe), and the scores ranged from 0 to 56 (Hamilton, 1959). HDRS scores > 8 indicated possible depression, >20 indicated depression; HARS scores > 7 indicated possible anxiety, >14 indicated anxiety.

#### Quality of Life Assessment

The McGill Quality of Life Questionnaire (MQOL), a 17-item [a single-item scale (MQOL-SIS) and 16 other items] multidimensional tool, was used to evaluate QOL in ALS patients. There are five MQOL subscales, which are designed to measure physical well-being, physical symptoms, psychological symptoms, existential well-being and social support, as well as overall quality of life. Each question uses a 0–10 scale. Each subscale score is the mean of the items forming that subscale. The MQOL Total (summary) score is the mean of the 5 subscale scores (Cohen et al., 1996, 1997).

The study was approved by the Research Ethics Committee of West China Hospital, Sichuan University (approval 2020-842). All participants were informed of the purpose of the study before enrollment and gave written informed consent for participation.

#### Statistical Analysis

Continuous parameters that were normally distributed were described as the means ± standard deviation (SD). Those with a non-normal distribution are presented as the median values. Categorical variables were summarized using patient counts and percentages. Student’s *t* test (with the Mann–Whitney *U* test when necessary) was used to compare continuous variables, and the chi-square test was used to analyze categorical variables.

The risk factors for pain in ALS patients were investigated by univariate regression analysis, and the results were controlled using a binary logistic regression model. Binary logistic regression analysis (forward stepwise LR method) was used to evaluate the association between the variables and pain. Variables with statistical significance (*p* value < 0.05) in the univariate regression analysis or considered clinically relevant were included in the multivariate analysis. The Box-Tidwell test was used for the assumption of linearity in the logit for the continuous variable. Multicollinearity was assessed using an examination of the variance inflation factor in a multiple regression model with the same variables. The results were expressed as odds ratios (ORs) with their corresponding 95% CIs (confidence intervals). The sample size in our study met the demands of events per variable (EPV) during logistic regression analysis.

Similarly, variables related to pain intensity and QoL were included in a multiple linear stepwise regression model. Data were tested for linearity, independence of residuals, homoscedasticity, normality, lack of multicollinearity, and no large leveraged values. A two-tailed *p* value < 0.05 was considered statistically significant. All statistical analyses were performed using SPSS 25.0.

## Results

### Demographic and Clinical Characteristics

A total of 86 ALS patients were included in the study, 50 men and 36 women, with a mean age of 52.6 years and a mean disease duration of 11 months. There were no significant differences in sex, age, age at onset, site of onset, disease duration, ALSFRS-R, ALSSS (LE + UE), ALSSS (SP + SW), or ALSSS (LE + UE + SP + SW) between ALS patients with pain and without pain (*p* > 0.05).

Thirty-eight ALS patients (44.2%) reported pain, with a mean PSI score of 3.2, among which thirty-three patients had a spinal onset. Nineteen males (38%) and nineteen females (52.8%) reported pain, with mean PSI scores of 2.9 and 3.5, respectively. Twenty patients (52.6%) had mild pain (1 ≤ PSI ≤ 3), thirteen patients (34.2%) had moderate pain (4 ≤ PSI ≤ 6), and only two patients (5.3%) had severe pain (7 ≤ PSI ≤ 10).

Compared with ALS patients without pain, ALS patients with pain were more likely to have higher FSS score (*p* = 0.023; Table 1). Additionally, the MQOL-Psychological, MQOL-Existential wellbeing, MQOL-Social support, and MQOL-Total scores were significantly lower in patients with pain than in patients without pain (*p* = 0.044, *p* = 0.001, *p* = 0.007, *p* = 0.030, respectively; Table 2).

**TABLE 1:** Demographic and clinical characteristics of amyotrophic lateral sclerosis (ALS) patients with pain or without pain.

	All ALS patients (*n* = 86)	ALS patients with pain (*n* = 38)	ALS patients without pain (*n* = 48)	*p* value
Gender (F: M)	36:50	19:19	17:31	0.173
Age at interview, year (mean ± SD)	52.6 ± 10.9	51.8 (10.1)	53.3 (11.5)	0.509
Age at onset, year (mean ± SD)	51.6 ± 11.0	50.8 (10.3)	52.2 (11.6)	0.555
Spinal onset, *n* (%)	68 (79.1)	33 (86.8)	35 (72.9)	0.115
Disease duration (range)	11 (2–84)	11.5 (2–84)	10.5 (2–72)	0.747
ALSFRS-R score (range)	40 (21–47)	41.5 (21–47)	40.0 (21–47)	0.731
ALSSS (LE + UE) (range)	19 (10–20)	20 (10–20)	18 (11–20)	0.076
ALSSS (SP + SW) (range)	16 (5–20)	16 (5–20)	16 (8–20)	0.807
ALSSS (LE + UE + SP + SW) (range)	34 (18–39)	35 (18–39)	32 (22–39)	0.297
HARS (range)	0–29 (6)	7.5 (0–29)	5 (0–24)	**0.042**
HARS>7, *n* (%)	37 (43)	19 (50)	18 (37.5)	0.245
HARS>14, *n* (%)	17 (19.8)	10 (26.3)	7 (14.6)	0.175
HDRS (range)	8 (0–30)	8.5 (0–28)	7.5 (0–30)	0.741
HDRS>8, *n* (%)	41 (47.7)	19 (50)	22 (45.8)	0.701
HDRS>20, *n* (%)	7 (8.1)	3 (7.9)	4 (8.3)	1.000
FSS (range)	2.4 (1–7)	3.2 (1–7)	2 (1–7)	**0.023**
FSS ≥ 4, *n* (%)	28 (32.6)	16 (42.1)	12 (25)	0.093

*F, female; M, male; SD, standard deviation; ALSFRS-R, Amyotrophic Lateral Sclerosis Functional Rating Scale-revised; ALSSS, Amyotrophic Lateral Sclerosis Severity Scale, SP, speech; SW, swallowing; LE, lower extremity; UE, upper extremity; HARS, Hamilton Anxiety Rating Scale; HDRS, Hamilton Depression Rating Scale; FSS, Fatigue Severity Scale. Bold values means values with statistical significance.*

**TABLE 2:** Interference of pain on quality of life in amyotrophic lateral sclerosis (ALS) patients with pain or without pain.

	ALS patients with pain (*n* = 38)	ALS patients without pain (*n* = 48)	*p* value
MQOL-SIS	6 (0–10)	7 (3–10)	0.070
MQOL-PhS	5.7 ± 2.6	5.8 ± 2.9	0.788
MQOL-PhWB	5 (0–10)	5 (0–10)	0.582
MQOL-PsyS (range)	5.6 (1.8–10)	7.1 (2–10)	**0.044**
MQOL-EWB (range)	5.2 (0–9.3)	7.3 (0–10)	**0.001**
MQOL-SS (range)	6.5 (0–10)	8 (0–10)	**0.007**
MQOL-Total	5.8 ± 1.6	6.5 ± 1.6	**0.030**

*MQOL, McGill Quality of Life Questionnaire; MQOL-SIS, MQOL single-item scale; MQOL-PhS, MQOL-physical symptoms; MQOL-PhWB, MQOL-Physical well-being; MQOL-PsyS, MQOL-Psychological symptoms; MQOL-EWB, MQOL-Existential wellbeing; MQOL-SS, MQOL- Social support. Bold values means values with statistical significance.*

A comparison of demographical and clinical data between ALS patients with pain and without pain is shown in Table 1.

### Factors Associated With Pain in Amyotrophic Lateral Sclerosis Patients

Hamilton Anxiety Rating Scale (HARS) and FSS scores were included in the binary logistic model. Multivariate analysis (Table 3) showed that only the FSS score [OR = 1.278, 95% CI (1.022, 1.600), *p* = 0.032] was associated with a higher risk of pain. The results for factors associated with pain in univariate regression analysis were presented in Supplementary Table 1.

**TABLE 3:** Factors associated with the presence of pain in the binary logistic analysis.

Covariates	B	OR	95% CI	*p* value
FSS	0.246	1.278	(1.022, 1.600)	**0.032**
Constant	−1.000	0.368	–	**0.018**

*FSS, Fatigue Severity Scale; OR, odds ratio; CI, confidence interval. Cox and Snell R Square and Nagelkerke R Square values was 0.055 and 0.073 in this Binary logistic regression analysis (forward stepwise LR method). Bold values means values with statistical significance.*

### Factors Associated With Pain Intensity in Amyotrophic Lateral Sclerosis Patients With Pain

Amyotrophic Lateral Sclerosis Functional Rating Scale-revised (ALSFRS-R), ALSSS (LE + UE), ALSSS (LE + UE + SP + SW) and HDRS scores were included in the multiple linear stepwise regression model to find the clinical variables associated with pain severity. The PSI score, which was not associated with ALSFRS-R, ALSSS (LE + UE + SP + SW) or HDRS scores, showed a negative correlation with the ALSSS (LE + UE) score [*p* = 0.003, 95% CI (−0.352, −0.080); Table 4]. The screening for factors associated with pain intensity in univariate regression analysis were shown in Supplementary Table 2.

**TABLE 4:** Factors associated with the intensity of pain in the multiple linear regression analysis.

Covariates	B	Beta	95% CI	*p* value
ALSSS (LE+UE)	−0.216	−0.473	(−0.352, −0.080)	**0.003**
Constant	6.431	–	(4.344, 8.517)	**<0.001**

*ALSSS, Amyotrophic Lateral Sclerosis Severity Scale; LE, lower extremity; UE, upper extremity; CI, confidence interval. R^2^ and adjusted R^2^ are 0.224 and 0.202 in this multiple linear regression. Bold values means values with statistical significance.*

### The Impact of Clinical Variables on Quality of Life in Amyotrophic Lateral Sclerosis Patients With Pain

The results of the effect of clinical variables on quality of life were shown in Table 5 by the multiple linear stepwise regression. The influence of clinical variables on various domains of quality of life were screened in univariate regression analysis and displayed in Supplementary Table 3.

**TABLE 5:** The impact of clinical variables on quality of life in the multiple linear regression.

	Variables	R^2^	adjusted R^2^	B	Beta	95% CI	*p* value
MQOL-SIS	ALSSS (LE+UE)	0.399	0.383	0.397	0.632	(0.232, 0.561)	** < 0.001**
	Constant			0.130		(−2.397, 2.657)	0.918
MQOL-PhS	HARS	0.508	0.480	–0.168	–0.453	(−0.274, −0.062)	**0.003**
	ALSSS (LE+UE)			0.248	0.358	(0.049, 0.446)	**0.016**
	Constant			3.552		(−0.099, 7.202)	0.056
MQOL-PhWB	Site of onset	0.272	0.231	–3.272	–0.370	(−5.912, −0.632)	**0.017**
	HDRS			–0.130	–0.302	(−0.259, −0.001)	**0.048**
	Constant			9.633		(7.085, 12.182)	** < 0.001**
MQOL-PsyS	HDRS	0.283	0.263	–0.192	–0.532	(−0.295, −0.089)	**0.001**
	Constant			7.862		(6.615, 9.109)	** < 0.001**
MQOL-EWB	HDRS	0.143	0.120	–0.113	–0.379	(−0.206, −0.020)	**0.019**
	Constant			6.223		(5.096, 7.350)	** < 0.001**
MQOL-SS	–			–	–	–	**−**
MQOL-Total	HDRS	0.452	0.420	–0.106	–0.454	(−0.175, −0.037)	**0.003**
	ALSSS (LE+UE)			0.140	0.317	(0.010, 0.271)	**0.036**
	Constant			4.710		(2.311, 7.109)	** < 0.001**

*MQOL, McGill Quality of Life Questionnaire; MQOL-SIS, MQOL single-item scale; MQOL-PhS, MQOL-physical symptoms; MQOL-PhWB, MQOL-Physical well-being; MQOL-PsyS, MQOL-Psychological symptoms; MQOL-EWB, MQOL-Existential wellbeing; MQOL-SS, MQOL-Social support; ALSSS, Amyotrophic Lateral Sclerosis Severity Scale; LE, lower extremity; UE, upper extremity; HARS, Hamilton Anxiety Rating Scale; HDRS, Hamilton Depression Rating Scale; CIs, confidence intervals. –, no variables were entered into the equation. Bold values means values with statistical significance.*

## Discussion

In our previous study, the prevalence, severity, site, type of pain and treatment, as well as the relationship between pain and clinical parameters, were reported in ALS patients and healthy controls (An et al., 2021). Consistent with previous results (An et al., 2021), pain was common in our ALS cohort, reported by 44.2%, and was higher than that in healthy controls (36%). Additionally, most patients (52.6%) considered the pain intensity to be mild (1 ≤ PSI ≤ 3), in accord with a previous study result conducted among a large sample of ALS patients (Edge et al., 2020). Although no significant difference was found, ALS patients with a spinal onset seemed to be more likely to report pain (Taga et al., 2019).

In our study, no significant differences were found between ALS patients with and without pain relative to sex, age at interview, age at onset, site at onset, disease duration, ALSFRS, ALSSS or HDRS score, in accordance with previous reports (Pizzimenti et al., 2013). However, compared with pain-free ALS patients, ALS patients with pain tended to report poorer QoL and higher FSS scores. A previous study found that ALS patients with pain had lower QoL scores, although the difference was not significant (Pizzimenti et al., 2013). Additionally, a significantly higher HAD anxiety score was found in ALS patients with pain than in those without pain (Lopes et al., 2018). However, no study has explored fatigue in ALS patients with pain thus far. As ALS progresses, atrophy and weakness of muscles and prolonged immobility cannot only cause degenerative changes in connective tissue, bones, and joints leading to musculoskeletal pain (Chiò et al., 2017) but also likely contribute to fatigue symptoms when moving, walking or exercising, which may account for the higher FSS score detected in ALS patients with pain. After multivariate regression analysis, only the FSS score was associated with the presence of pain in ALS patients, further proving the relationship between the FSS score and a higher risk of pain.

Regarding the intensity of pain, previous studies found there was a weak negative correlation between the ALSFRS-R and PSI score (Chiò et al., 2012; Hanisch et al., 2015; Moisset et al., 2016; An et al., 2021); additionally, there was no significant correlation between pain intensity and disease duration or depression scores (Pizzimenti et al., 2013; Hanisch et al., 2015; Moisset et al., 2016; An et al., 2021). On the contrary, pain intensity was reported to be statistically correlated with anxiety and depression in Brazilian patients, measured by the Hospital Anxiety and Depression Scale (HADS). Different outcome measures for depression and anxiety among various ethnic groups between studies may be responsible for the different findings (Lopes et al., 2018). In our study, ALSSS (LE + UE) was found to be negatively associated with the intensity of pain. ALSSS (LE + UE) score was representative of the functional status of limbs in patients with ALS. Also, just as mentioned above, dysfunction of limbs, which usually resulted from muscle weakness and atrophy, may cause musculoskeletal pain in ALS patients. Therefore, pain intensity may be most affected by the ALSSS (LE + UE) score.

A previous study indicated that ALS patients described their QoL as involving physical, psychological and other aspects (Edge et al., 2020). Considering both the physical and psychological aspects of QoL in ALS patients jointly facilitated a greater understanding of how clinical variables affect QoL. A multidimensional tool, the MQOL questionnaire, was adopted to assess QoL in ALS patients. The QOL questionnaire has been widely used in ALS patients (Simmons et al., 2000; Robbins et al., 2001; Lou et al., 2003, 2010; Chiò et al., 2004). Here, we found that ALSSS (LE + UE), HARS, and HDRS scores were significantly related to QoL, affecting physical symptoms, physical well-being, psychological symptoms and existential wellbeing domains, as well as overall quality of life.

In our study, there was no significant effects of pain intensity on different aspects of QoL in ALS patients, consistent with the findings of some former studies (Pizzimenti et al., 2013; Lopes et al., 2018). In contrast, a previous work in Italy, in which the same MQOL questionnaire was used for QoL (Pagnini et al., 2012), suggested that pain was significantly correlated with overall QoL, physical symptoms and existential wellbeing but not psychological wellbeing. However, that sample size was small, and only thirty-one patients with ALS were ultimately included; then, the relationship between pain and QoL was evaluated, without considering any other potentially influencing or confounding factors, such as depression. Also, pain was reported to have a significant effect on the physical domain of QoL, in which another different multidimensional measuring scale for QoL was adopted (Edge et al., 2020). Another study found a weak association between increasing pain and lower QoL, which was no longer statistically significant once depression was accounted for Pizzimenti et al. (2013). Additionally, only a total of 36 ALS patients were included, and the QoL measure used was the Spitzer QoL index, which was reported as a single number between 0 and 10 (Pizzimenti et al., 2013). Therefore, using a multidimensional description of QoL in our study and including other potentially influencing or confounding factors in our analysis allowed a more comprehensive and reliable depiction of QoL related to pain.

Quality of life (QoL) was found to be negatively affected by depression score in ALS patients, including MQOL-physical well-being, psychological symptoms, existential wellbeing domains and MQOL-Total. Previous studies have also reported that depression scores can significantly decrease QoL (Lou et al., 2003; Kübler et al., 2005; Lulé et al., 2008; Tramonti et al., 2012; Pizzimenti et al., 2013; Sandstedt et al., 2016; Lopes et al., 2018; Edge et al., 2020) in both physical and psychological domains (Edge et al., 2020). Additionally, only three ALS patients with pain (7.9%) exceeded the upper limit of range for depression diagnosis in our sample, suggesting that those with subthreshold scores for depressive symptoms should be actively treated due to their negative effect on the QoL of ALS patients (Pizzimenti et al., 2013).

Likewise, anxiety score seriously affected MQOL-physical symptoms of QoL. Prior studies also indicated that anxiety can undermine quality of life in ALS patients (Vignola et al., 2008; Lopes et al., 2018). One study, consisting of sixty ALS patients, found that depression and anxiety, assessed by the HADS, were associated with the psychosocial score rather than the physical score in the Sickness Impact Profile measure for QoL (Sandstedt et al., 2016). Another study with a much larger sample of ALS patients reported that anxiety was mildly correlated with physical QoL (Edge et al., 2020). Considering the MQOL-physical symptoms domain indicated the degrees to which the most three miserable physical symptoms caused distress and there was a close relationship between the severity of physical symptoms and mood in patients, not surprisingly, the more serious the MQOL-physical symptoms were, the higher the HARS score.

Functional status (ALSFRS) was reported to be significantly correlated with QoL measured by the MQOL-SIS (Lou et al., 2010) or ALSAQ-40 (Lopes et al., 2018). In our study, we found there was significant impact of functional status of the limbs on different domains of QoL (including MQOL-SIS, MQOL-physical symptoms and MQOL-Total), which were measured by ALSSS (LE + UE) and MQOL scales, respectively. Different scales used for the measurement in various ethnic populations may account for the difference between studies.

There were some strong strengths in our study that should be noted. First, this was the first study to comprehensively investigate factors associated with pain in Chinese ALS patients and found that fatigue can be a risk factor for the presence of pain and ALSSS (LE + UE) score was related with pain intensity, which has never been reported worldwide. Second, considering that ALS patients described their QoL as involving physical, psychological and other aspects, we innovatively used a multidimensional MQOL scale to comprehensively evaluate the clinical symptoms on quality of life (physical, psychological social support, and existential factors) in ALS patients. Lastly, besides disease duration, disease severity and depression, which were the common variables included in previous studies, we added other potential factors, such as anxiety, sleep and fatigue in our study, and all the assessments were conducted by using standard specialized measures.

The limitations of our work were as follows: first, the sample size was relatively small, and only two variables (HARS, FSS score) could be included in the binary logistic regression analysis. Further studies with a larger sample size of ALS patients from different ethnic populations could be conducted to confirm our findings. Then, we analyzed and found factors associated with pain and pain intensity; however, the impact of the nature of pain, type of pain, duration and frequency of pain were not included in our analysis. Last, our study was a cross-sectional design without follow-up, and we could not determine whether the effects on QoL varied over time or whether effective treatments for limb dysfunction, anxiety and depression symptoms could improve QoL. Concerning this disease being uncurable now, limb and mood disturbances offer another potentially important treatment target for improving QoL. Whether effective management of those symptoms can help improve various aspects of QoL in ALS patients deserves further study.

## Conclusion

In conclusion, our study found that fatigue may be a potential risk factor for pain and ALSSS (LE + UE) score was related with pain intensity in Chinese ALS patients. Additionally, it highlighted the adverse effects of ALSSS (LE + UE), anxiety and depressive symptom scores on various domains of QoL. Accordingly, clinicians should routinely pay more attention to limbs dysfunction, anxiety and depressive symptoms in ALS patients and treat them in a timely and appropriate manner.

## Data Availability Statement

The original data of the current study are available from the corresponding author on reasonable request.

## Ethics Statement

This study was reviewed and approved by the Institutional Ethics Committee of West China Hospital, Sichuan University (approval 2020-842). The patients/participants provided their written informed consent to participate in this study.

## Author Contributions

All authors contributed to the study conception and design. RA, YW, YL, XL, and SA performed material preparation, data collection, and analysis. RA wrote the first draft of the manuscript. CH and YX designed the research project and revised the manuscript. All authors commented on previous versions of the manuscript, read, and approved the final manuscript.

## Conflict of Interest

The authors declare that the research was conducted in the absence of any commercial or financial relationships that could be construed as a potential conflict of interest.

## Publisher’s Note

All claims expressed in this article are solely those of the authors and do not necessarily represent those of their affiliated organizations, or those of the publisher, the editors and the reviewers. Any product that may be evaluated in this article, or claim that may be made by its manufacturer, is not guaranteed or endorsed by the publisher.
