# Supplementary material for: Pain-Related Factors and Their Impact on Quality of Life in Chinese Patients With Amyotrophic Lateral Sclerosis
Source: Front Neurosci. 2022 Jul 13;16:897598. doi: 10.3389/fnins.2022.897598 (PMC9340542; doi:10.3389/fnins.2022.897598)
Supplement: Supplementary file 1 [file Table_1.docx]

**Supplement table 1** **Factors associated with the presence of pain in univariate regression analysis**

|  | **B** | **OR** | **95% CI** | **p value** |
| --- | --- | --- | --- | --- |
| Gender | -0.601 | 0.548 | (0.230, 1.307) | 0.175 |
| Age at interview | -0.014 | 0.987 | (0.948, 1.027) | 0.504 |
| Age at onset | -0.012 | 0.988 | (0.950, 1.028) | 0.551 |
| Site of onset | 0.897 | 2.451 | (0.787, 7.633) | 0.122 |
| Disease duration | -0.002 | 0.998 | (0.969, 1.029) | 0.911 |
| ALSFRS-R score | 0.005 | 1.005 | (0.937, 1.078) | 0.882 |
| ALSSS(LE+UE) | -0.031 | 0.970 | (0.855, 1.100) | 0.634 |
| ALSSS(SP+SW) | 1.117 | 1.124 | (0.975, 1.296) | 0.107 |
| ALSSS (LE+UE+SP+SW) | 0.037 | 1.038 | (0.945, 1.140) | 0.440 |
| **HARS** | 0.069 | 1.071 | (0.999, 1.148) | **0.054** |
| HDRS | 0.008 | 1.008 | (0.950, 1.071) | 0.784 |
| **FSS** | 0.027 | 1.028 | (1.002, 1.054) | **0.032** |

ALSFRS-R: Amyotrophic Lateral Sclerosis Functional Rating Scale-revised; ALSSS: Amyotrophic lateral sclerosis severity scale, SP: speech; SW: swallowing; LE: lower extremity; UE: upper extremity. HARS: Hamilton Anxiety Rating Scale; HDRS: Hamilton Depression Rating Scale; FSS: Fatigue Severity Scale. OR: Odds ratio; CI: confidence interval. Clinical variables in bold with statistical significance in univariate regression analysis or considered clinically relevant were included in the multivariate analysis.
