# Supplementary material for: Pain-Related Factors and Their Impact on Quality of Life in Chinese Patients With Amyotrophic Lateral Sclerosis
Source: Front Neurosci. 2022 Jul 13;16:897598. doi: 10.3389/fnins.2022.897598 (PMC9340542; doi:10.3389/fnins.2022.897598)
Supplement: Supplementary file 2 [file Table_2.docx]

**Supplement table 2  Factors associated with the intensity of pain in the univariate regression analysis**

| [**Independent**](javascript:;) [**variable**](javascript:;) | **B** | **OR** | **95% CI** | **p value** |
| --- | --- | --- | --- | --- |
| Gender | -0.632 | -0.190 | (-1.737, 0.474) | 0.254 |
| Age at interview | -0.004 | -0.023 | (-0.061, 0.053) | 0.889 |
| Age at onset | -0.003 | -0.021 | (-0.059, 0.052) | 0.901 |
| Site of onset | 1.087 | 0.221 | (-0.538, 2.711) | 0.183 |
| Disease duration | 0.005 | 0.040 | (-0.035, 0.044) | 0.812 |
| **ALSFRS-R score** | -0.086 | -0.322 | (-0.171, -0.001) | **0.048** |
| **ALSSS(LE+UE)** | -0.216 | -0.473 | (-0.352, -0.080) | **0.003** |
| ALSSS(SP+SW) | -0.018 | -0.033 | (-0.207, 0.171) | 0.845 |
| **ALSSS (LE+UE+SP+SW)** | -0.134 | -0.383 | (-0.243, -0.025) | **0.018** |
| HARS | 0.048 | 0.040 | (-0.033, 0.129) | 0.238 |
| **HDRS** | 0.061 | 0.256 | (-0.017, 0.140) | **0.121** |
| FSS | 0.014 | 0.153 | (-0.017, 0.046) | 0.359 |

ALSFRS-R: Amyotrophic Lateral Sclerosis Functional Rating Scale-revised; ALSSS: Amyotrophic lateral sclerosis severity scale, SP: speech; SW: swallowing; LE: lower extremity; UE: upper extremity. HARS: Hamilton Anxiety Rating Scale; HDRS: Hamilton Depression Rating Scale; FSS: Fatigue Severity Scale. OR: Odds ratio; CI: confidence interval. Clinical Variables in bold with statistical significance in univariate regression analysis or considered clinically relevant were included in the multivariate analysis.
