# Supplementary material for: Pain-Related Factors and Their Impact on Quality of Life in Chinese Patients With Amyotrophic Lateral Sclerosis
Source: Front Neurosci. 2022 Jul 13;16:897598. doi: 10.3389/fnins.2022.897598 (PMC9340542; doi:10.3389/fnins.2022.897598)
Supplement: Supplementary file 3 [file Table_3.docx]

**Supplement table 3  Factors associated with quality of life in the univariate regression analysis**

|  |  | **B** | **OR** | **95% CI** | **p value** |
| --- | --- | --- | --- | --- | --- |
| MQOL-SIS | Site of onset | -1.321 | -0.195 | (-3.571, 0.928) | 0.241 |
|  | Disease duration | -0.001 | -0.008 | (-0.055, 0.053) | 0.964 |
|  | **ALSFRS-R score** | 0.244 | 0.640 | (0.152, 0.336) | **<0.001** |
|  | **ALSSS(LE+UE)** | 0.405 | 0.646 | (0.255, 0.556) | **<0.001** |
|  | ALSSS(SP+SW) | 0.124 | 0.155 | (-0.126, 0.375) | 0.322 |
|  | **ALSSS (LE+UE+SP+SW)** | 0.297 | 0.603 | (0.173, 0.421) | **<0.001** |
|  | **HARS** | -0.165 | -0.557 | (-0.243, -0.088) | **<0.001** |
|  | **HDRS** | -0.150 | -0.537 | (-0.224, -0.075) | **<0.001** |
|  | **PSQI** | -0.197 | -0.423 | (-0.331, -0.064) | **0.005** |
|  | **FSS** | -0.348 | -0.297 | (-0.725, 0.030) | **0.070** |
|  | PSI | -0.206 | -0.150 | (-0.667, 0.254) | 0.369 |
| MQOL-PhS | Site of onset | -1.469 | -0.196 | (-3.947, 1.010) | 0.237 |
|  | Disease duration | 0.006 | 0.032 | (-0.054, 0.065) | 0.851 |
|  | **ALSFRS-R score** | 0.238 | 0.589 | (0.128, 0.349) | **<0.001** |
|  | **ALSSS(LE+UE)** | 0.417 | 0.602 | (0.230, 0.604) | **<0.001** |
|  | ALSSS(SP+SW) | 0.103 | 0.121 | (-0.182, 0.387) | 0.468 |
|  | **ALSSS (LE+UE+SP+SW)** | 0.285 | 0.537 | (0.134, 0.436) | **0.001** |
|  | **HARS** | -0.240 | -0.646 | (-0.336, -0.144) | **<0.001** |
|  | **HDRS** | -0.200 | -0.548 | (-0.303, -0.097) | **<0.001** |
|  | **PSQI** | -0.307 | -0.581 | (-0.453, -0.161) | **<0.001** |
|  | **FSS** | -0.540 | -0.418 | (-0.935, -0.144) | **0.009** |
|  | PSI | -0.467 | -0.308 | (-0.955, 0.021) | 0.060 |
| MQOL-PhWB | **Site of onset** | -3.800 | -0.430 | (-6.499, -1.101) | **0.007** |
|  | Disease duration | -0.017 | -0.083 | (-0.088, 0.053) | 0.619 |
|  | **ALSFRS-R score** | 0.147 | 0.307 | (-0.007, 0.301) | **0.060** |
|  | **ALSSS(LE+UE)** | 0.342 | 0.418 | (0.091, 0.594) | **0.009** |
|  | ALSSS(SP+SW) | -0.092 | -0.092 | (-0.429, 0.246) | 0.585 |
|  | ALSSS (LE+UE+SP+SW) | 0.165 | 0.263 | (-0.039, 0.370) | 0.110 |
|  | **HARS** | -0.125 | -0.284 | (-0.267, 0.018) | **0.084** |
|  | **HDRS** | -0.162 | -0.375 | (-0.297, -0.027) | **0.020** |
|  | PSQI | -0.076 | -0.122 | (-0.286, 0.134) | 0.467 |
|  | FSS | -0.025 | -0.017 | (-0.541, 0.490) | 0.921 |
|  | **PSI** | -0.680 | -0.379 | (-1.241, -0.118) | **0.019** |
| MQOL-PsyS | Site of onset | -0.794 | -0.107 | (-3.276, 1.689) | 0.521 |
|  | Disease duration | -0.033 | -0.188 | (-0.091, 0.025) | 0.258 |
|  | ALSFRS-R score | 0.064 | 0.160 | (-0.069, 0.197) | 0.337 |
|  | ALSSS(LE+UE) | 0.162 | 0.236 | (-0.063, 0.386) | 0.153 |
|  | ALSSS(SP+SW) | -0.145 | -0.174 | (-0.424, 0.133) | 0.297 |
|  | ALSSS (LE+UE+SP+SW) | 0.038 | 0.073 | (-0.139, 0.215) | 0.665 |
|  | **HARS** | -0.175 | -0.476 | (-0.284, -0.066) | **0.003** |
|  | **HDRS** | -0.192 | -0.532 | (-0.295, -0.089) | **0.001** |
|  | **PSQI** | -0.198 | -0.378 | (-0.361, -0.034) | **0.019** |
|  | **FSS** | -0.523 | -0.411 | (-0.916, -0.130) | **0.010** |
|  | PSI | 0.016 | 0.010 | (-0.491, 0.522) | 0.950 |
| MQOL-EWB | **Site of onset** | -2.028 | -0.332 | (-3.974, -0.082) | **0.042** |
|  | Disease duration | 0.012 | 0.080 | (-0.037, 0.060) | 0.631 |
|  | **ALSFRS-R score** | 0.105 | 0.318 | (-0.001, 0.211) | **0.052** |
|  | **ALSSS(LE+UE)** | 0.175 | 0.309 | (-0.007, 0.356) | **0.059** |
|  | ALSSS(SP+SW) | -0.075 | -0.108 | (-0.307, 0.158) | 0.518 |
|  | ALSSS (LE+UE+SP+SW) | 0.073 | 0.169 | (-0.071, 0.218) | 0.310 |
|  | **HARS** | -0.106 | -0.350 | (-0.202, -0.010) | **0.031** |
|  | **HDRS** | -0.113 | -0.379 | (-0.206, -0.020) | **0.019** |
|  | **PSQI** | -0.094 | -0.219 | (-0.237, 0.048) | **0.187** |
|  | FSS | -0.078 | -0.074 | (-0.432, 0.277) | 0.660 |
|  | **PSI** | -0.318 | -0.257 | (-0.723, 0.087) | **0.120** |
| MQOL-SS | Site of onset | -0.645 | -0.109 | (-2.628, 1.337) | 0.513 |
|  | Disease duration | 0.009 | 0.065 | (-0.038, 0.056) | 0.698 |
|  | ALSFRS-R score | 0.081 | 0.254 | (-0.023, 0.186) | 0.124 |
|  | ALSSS(LE+UE) | 0.114 | 0.208 | (-0.067, 0.294) | 0.210 |
|  | ALSSS(SP+SW) | -0.006 | -0.010 | (-0.233, 0.220) | 0.955 |
|  | ALSSS (LE+UE+SP+SW) | 0.064 | 0.154 | (-0.076, 0.204) | 0.357 |
|  | HARS | -0.030 | -0.101 | (-0.128, 0.069) | 0.546 |
|  | HDRS | -0.051 | -0.177 | (-0.147, 0.045) | 0.288 |
|  | PSQI | -0.024 | -0.057 | (-0.164, 0.117) | 0.736 |
|  | FSS | -0.183 | -0.180 | (-0.521, 0.156) | 0.280 |
|  | PSI | -0.085 | -0.071 | (-0.489, 0.319) | 0.671 |
| MQOL-Total | **Site of onset** | -1.747 | -0.365 | (-3.255, -0.240) | **0.024** |
|  | Disease duration | -0.005 | -0.042 | (-0.043, 0.033) | 0.800 |
|  | **ALSFRS-R score** | 0.127 | 0.490 | (0.051, 0.204) | **0.002** |
|  | **ALSSS(LE+UE)** | 0.242 | 0.545 | (0.116, 0.367) | **<0.001** |
|  | ALSSS(SP+SW) | -0.043 | -0.079 | (-0.226, 0.140) | 0.636 |
|  | **ALSSS (LE+UE+SP+SW)** | 0.125 | 0.368 | (0.018, 0.232) | **0.023** |
|  | **HARS** | -0.135 | -0.567 | (-0.201, -0.069) | **<0.001** |
|  | **HDRS** | -0.143 | -0.614 | (-0.206, -0.081) | **<0.001** |
|  | **PSQI** | -0.140 | -0.412 | (-0.244, -0.035) | **0.010** |
|  | **FSS** | -0.270 | -0.326 | (-0.534, -0.006) | **0.045** |
|  | PSI | -0.307 | -0.316 | (-0.619, 0.005) | 0.054 |

ALSFRS-R: Amyotrophic Lateral Sclerosis Functional Rating Scale-revised; ALSSS: Amyotrophic lateral sclerosis severity scale, SP: speech; SW: swallowing; LE: lower extremity; UE: upper extremity. HARS: Hamilton Anxiety Rating Scale; HDRS: Hamilton Depression Rating Scale; PSQI: Pittsburgh Sleep Quality Index; FSS: Fatigue Severity Scale. PSI: Pain Severity Index; MQOL: McGill Quality of Life Questionnaire; MQOL-SIS: MQOL single-item scale; MQOL-PhS: MQOL-physical symptoms; MQOL-PhWB: MQOL-Physical well-being; MQOL-PsyS: MQOL-Psychological symptoms; MQOL-EWB: MQOL-Existential wellbeing; MQOL-SS: MQOL-Social support. OR: Odds ratio; CI: confidence interval. Clinical Variables in bold with statistical significance in univariate regression analysis or considered clinically relevant were included in the multivariate analysis.
